# Non-curative surgery for patients with gastric cancer with local peritoneal metastasis: A retrospective cohort study

**DOI:** 10.1097/MD.0000000000005607

**Published:** 2016-12-09

**Authors:** Yuanqiang Dong, Shulan Ma, Shuo Yang, Fen Luo, Zhiming Wang, Fenghua Guo

**Affiliations:** a Department of General Surgery, Huashan Hospital; b Department of Gynecology, Huashan North Hospital; c Central Laboratory, Huashan North Hospital; d Surgical Laboratory, Huashan Hospital, Shanghai Medical College, Fudan University, Shanghai, China.

**Keywords:** gastric cancer, non-curative surgery, peritoneal metastasis

## Abstract

The role of non-curative surgery for patients with M1 gastric cancer (GC) is controversial. This study aimed to evaluate the efficacy of non-curative resectional surgery for patients with GC with local peritoneal metastasis.

We reviewed the medical records of 47 patients with GC with local peritoneal metastasis, which was found by laparotomy or laparoscopy. The patients were divided into 2 groups: those who underwent gastric resection (n = 29), and a non-resection group who did not (n = 18). The clinical characteristics, postoperative complications, mortality, palliative intervention, and long-term outcomes of the 2 groups were compared.

Complications occurred more frequently in the resection group than in non-resection group (*P* = 0.017). There was no postoperative mortality or reoperation in either group. Palliative intervention was performed in 9 (31%) patients in resection group and 16 (88.9%) patients in non-resection group (*P* < 0.001). The intervention interval and hospital-free time were significant longer in resection group than in non-resection group (*P* < 0.001, *P* < 0.001). The Kaplan–Meier survival curves revealed that resection group had longer survival than non-resection group (*P* < 0.001).

Non-curative resectional surgery helps prolong survival time and improve the quality of life for patients with GC with local peritoneal metastasis.

## 1 Introduction

Despite recent advances in diagnosis and treatment, gastric cancer (GC) remains one of the most common cancers.^[1]^ Based on the data from 2008, around 980,000 new GC cases were diagnosed and 0.74 million GC deaths occurred worldwide.^[2]^ In China, more than 0.4 million new GC cases are diagnosed yearly.^[3,4]^ The prognosis of those patients is still very poor, even after chemoradiotherapy and target therapy.^[5]^ It was reported that non-curative resection might prolong survival time.^[6–12]^ However, other studies had contradictory results.^[13–16]^ Most of the studies on the effects of non-curative resection are retrospective, and lack clear selection criteria and stratification. Selective bias and variability in tumor burden and performance status present controversy about the role of non-curative resection in incurable patients with GC.

In this retrospective study, we aimed to clarify the efficacy of non-curative surgery in patients with incurable GC with local peritoneal metastasis.

## 2 Methods

### 2.1 Patients

A total of 3108 patients diagnosed with gastric carcinoma by pathological biopsy at Huashan Hospital affiliated to Fudan University between January 2002 and December 2012 were screened. The inclusion criteria were: no extra abdominal metastasis before operation; only peritoneal metastases were found by laparotomy or laparoscopy; peritoneal metastasis limited to the area above the transverse colon (including lesser sac, lesser omenta, greater omenta, diaphragm, and peritoneum around the liver and spleen) and less than 1 cm in diameter; and after surgical exploration, patients were recommended for non-curative resection. Finally, the clinical data of 47 patients with local peritoneal metastasis were analyzed in this study. According to the patient's preference, 29 patients underwent non-curative resection, the other 18 patients did not undergo resection surgery. Non-curative resectional surgery was defined as subtotal or total gastrectomy and D1 or D2 lymphadenectomy, but with a postoperative residual disease according to Japanese GC treatment guidelines.^[17]^ In resection group, greater omenta, lesser omenta, and lesser sac were routinely resected, no peritoneum more than the scope of radical gastrectomy were removed. All 47 patients were recommended for postoperative chemotherapy and intraperitoneal chemotherapy.

### 2.2 Clinical data collection

Postoperative intervention was defined as all procedures performed for the relief of gastrointestinal obstruction, bleeding from tumor, biliary obstruction, ascites, and other symptoms caused by GC or metastasis. Hospital-free time (HFT) was defined as the time from the discharge after operation to the death or rehospitalization for more than 1 month. Intervention interval was defined as the time from the operation to the first palliative intervention.

The following clinical data were extracted and analyzed: demographic findings, extent of gastric resection, extent of lymphadenectomy, postoperative complications, postoperative mortality, postoperative chemotherapy, intraperitoneal chemotherapy, HFT, postoperative intervention, intervention interval, and long-term outcomes. No ethical approval or patient consent was required because this was a retrospective study.

### 2.3 Statistical analysis

Statistical analysis was performed using the SPSS 18.0. Collected data were expressed as medians, frequencies, percentages, and mean ± SD. The χ^2^ test or Fisher exact test and Student *t*-test were used for the comparison. Overall survival (OS) was calculated from the time of operation to the date of death or the most recent follow-up. Survival curves were estimated using the Kaplan–Meier method and compared by the log-rank test. The χ^2^ test or Fisher exact test, and Student *t*-test were two-sided. Statistical significance was set at *P* < 0.05.

## 3 Results

### 3.1 Clinical characteristics and surgical outcomes

Clinical characteristics and surgical outcomes of the patients are shown in Table 1. There were no significant differences in the age, sex, and tumor location between resection group and non-resection group (*P* = 0.108, *P* = 0.474, *P* = 0.95, respectively). The extent of gastric resection was selected by the location and size of the lesions, and the extent of lymphadenectomy was determined by the surgeon. In resection group, 12 patients underwent subtotal gastrectomy and 17 underwent total gastrectomy; D1 and D2 lymphadenectomy was performed in 5 and 24 patients, respectively. Postoperative complications developed in 11 of 29 patients in resection group and 1 patient in non-resection group, including wound infection, postoperative bleeding, urinary tract infections, and pneumonia. Complications occurred more frequently in resection group (*P* = 0.017). There was no postoperative mortality and complication-related reoperation in both groups.

**Table 1:**
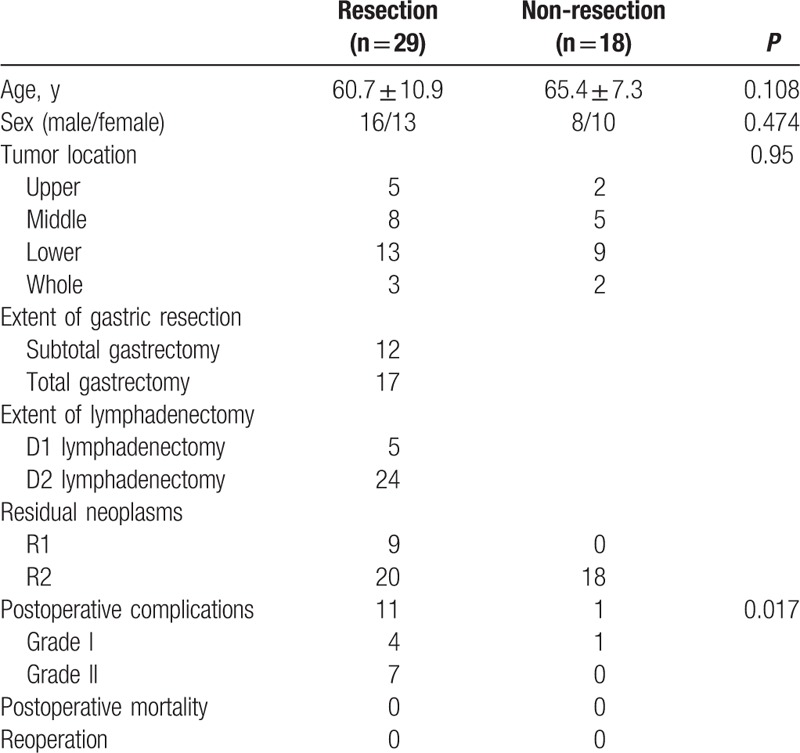
Patients’ clinical outcome.

### 3.2 Postoperative chemotherapy and intervention

All patients received postoperative chemotherapy (Table 2). In comparison, 24 patients in resection group and 11 patients in non-resection group received paclitaxel or cis-platinum based intraperitoneal chemotherapy (*P* = 0.168).

**Table 2:**
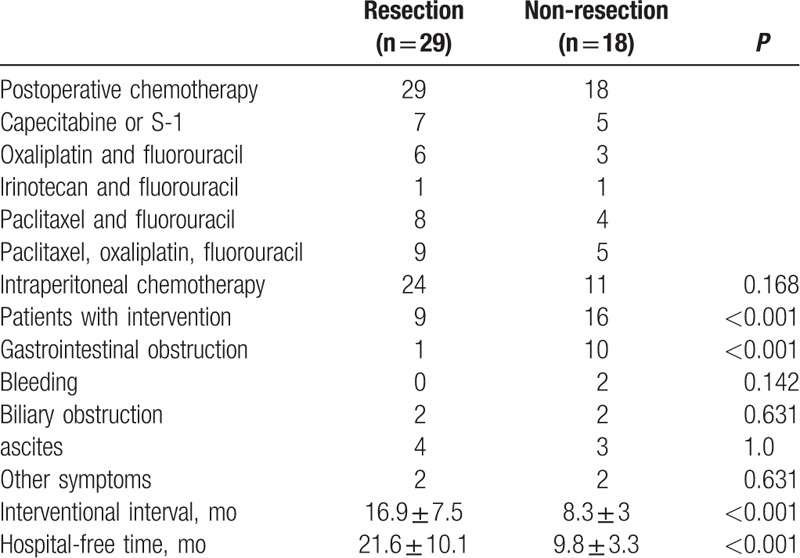
Postoperative chemotherapy and intervention.

Palliative intervention was performed on 9 (31%) patients in resection group and 16 (88.9%) patients in non-resection group (*P* < 0.001, Table 2). The interventional interval in resection group was 16.9 ± 7.5 months, significantly longer than 8.3 ± 3 months in non-resection group (*P* < 0.001). Most of those patients were relieved of symptoms through endoscopy and paracentesis, only 2 patients needed operation to relieve them of intestine obstruction caused by metastasis. There was no intervention-related death.

### 3.3 Survival

**Figure 1:**
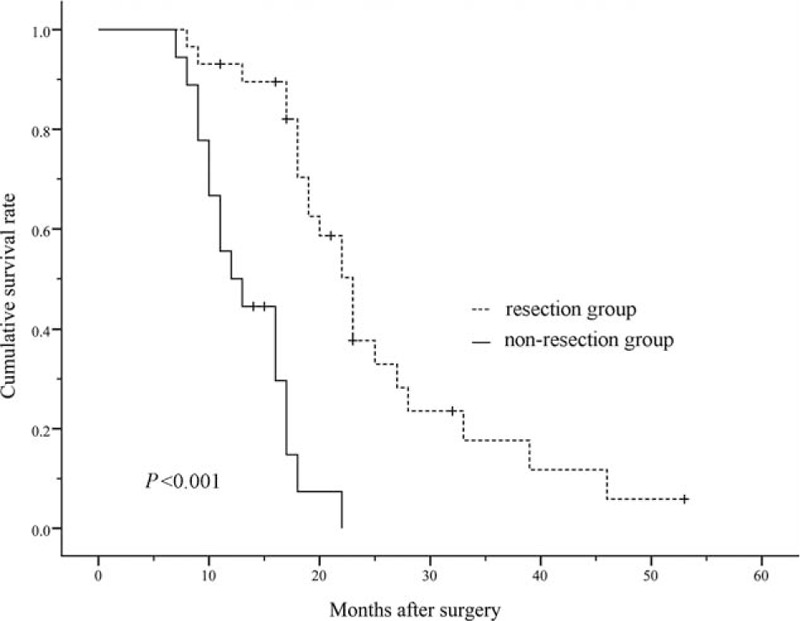
Kaplan–Meier survival curve analysis. The patients in resection group had longer survival than those in non-resection group (*P* < 0.001).

In resection group, with a median follow-up of 22 months (range, 8–53 months), 1-year, 2-year, 3-year OS rates were 93.1%, 37.7%, 17.7%, respectively. In non-resection group, with a median follow-up of 12 months (range, 7–22 months), 1-year OS rate was only 50% (*P* = 0.001), and there was no 2-year survival. Median OS was 23 months in resection group and 12 months in non-resection group (*P* < 0.001). The Kaplan–Meier survival curves revealed that resection group have longer survival than non-resection group (*P* < 0.001, Fig. 1).

## 4 Discussion

In recent decades, many studies suggested that non-curative gastrectomy could prolong the survival time of patients with incurable disease.^[6–12]^ In those studies, selection bias existed in metastasis type, tumor burden, performance status, and adjuvant therapy, the median survival following non-curative surgery ranged from 3 to 24 months.^[18]^ However, other studies showed that non-curative surgery reduced the quality of life and could not prolong the survival time.^[13–16]^ Therefore, whether non-curative surgery is a viable therapeutic option for metastatic GC and which patients will benefit from the surgery remain unclarified. Randomized trials to address this issue do not exist and will likely not occur. So, it is exercisable to evaluate retrospective data from record such as this to discuss the value of non-curative surgery.

In our hospital, best supportive care and chemotherapy are recommended to almost all patients with GC with preoperative metastasis. If organ metastasis or diffuse peritoneal metastasis is not found before operation but is confirmed intraoperatively, surgeons tend to terminate the operation, because the value of non-curative surgery in those patients was limited. Only in some patients with local peritoneal metastasis intraoperatively, and with good performance status and resectable tumor, the non-curative resection may be recommended. Patients receiving such recommendation were enrolled in the present study. However, the decision whether to perform a non-curative gastric resection or not was taken by the patient's family members during the operation. In other words, not medical factors but the family, social, and economic factors affect the choice of family members. The patients in the present study represent a high selective series, and the above treatment and decision-making process help reduce selection bias. The relatively low selection bias in this study helps determine the role of non-curative surgery for patients with GC with local peritoneal metastasis.

To evaluate the role of non-curative surgery, the first thing to consider is the balance between the operational risk and survival benefit. In this study, the complication rate was significantly higher in resection group (37.9%) than in non-resection group (5.5%). Most of the complications were pneumonia, wound infection, and urinary tract infections, so there was no complication-related reoperation and postoperative mortality in both groups. According to previous reports, postoperative morbidity and mortality incidence after radical surgery for GC were in the range of 12 to 46% and 2.2 to 13%, respectively.^[19–21]^ These results indicate that the incidence of complications and mortality in this study is not higher than that after radical surgery for GC. In this study, the patients in resection group had higher OS rate and longer median survival time than those in non-resection group. According to a recent meta-analysis about non-curative surgery in patients with GC, the range of median survival time was 3 to 24 months in resection group, and 4.8 to 12 months in non-resection group.^[16]^ However, the median survival time in both groups (23 months and 12 months) in this study is longer than that in most of previous studies. These satisfactory results can be partly explained by advanced surgical techniques. Moreover, we speculated that it is due to the application of intraperitoneal chemotherapy. According to recent reports, intraperitoneal chemotherapy helps control peritoneal metastasis, especially those small peritoneal metastases.^[22–24]^ These findings indicate that better physical condition, more local and smaller peritoneal metastases, and intraperitoneal chemotherapy are important factors related to lower surgical risk and longer median survival time, and non-curative surgery is safe and valuable in suitable patients with GC with local peritoneal metastases.

The quality of life and satisfactory palliative intervention are important for patients with stage IV GC. It is difficult to retrospectively assess the quality of life and the symptom improvement. Thus, in this study we used 2 alternatives, HFT and intervention interval. Nine patients in resection group and 16 patients in non-resection group underwent postoperative intervention to manage symptoms caused by primary tumor or metastasis. Consistent with previous study,^[25]^ gastrointestinal obstruction was the most common symptom in this series of patients. More patients in non-resection group needed palliative intervention to relieve gastrointestinal obstruction and to control bleeding from primary tumor. There was no difference between 2 groups in intervention to relieve other symptoms, including biliary obstruction, ascites, and headaches. These data suggest that resection of the primary tumor could reduce the incidence of gastrointestinal obstruction and bleeding. With the development of endoscopic and interventional techniques, some studies suggested that preemptive palliative gastrectomy in patients with stage IV GC should be avoided.^[25,26]^ In this study, most symptoms caused by obstruction and bleeding could be alleviated through endoscopy or paracentesis, but more patients in non-resection group received more than one intervention, and both HFT and intervention interval were significantly longer in resection group than in non-resection group. Taken together, these results suggest that patients in resection group have better quality of life.

However, several limitations of this study should be pointed point. First, our sample size is relatively small (<50). Second, the selection of the patients may be biased based on our inclusion and exclusion criteria. Third, our study is of retrospective nature. Further studies are needed to confirm that non-curative surgery is helpful to prolong survival time and improve the quality of life for high selective patients with GC with local peritoneal metastasis.

## Acknowledgments

We thank Dr Xiaobo Li, Department of Physiology and Pathophysiology, Fudan University Shanghai Medical College, Shanghai, and Shengmei Feng, Shanghai Roche Pharmaceuticals Ltd, for their constructive comments.
